# Endothelial cell-released mitochondrial DNA promotes B cell differentiation and virus replication during severe fever with thrombocytopenia syndrome virus infection

**DOI:** 10.1128/jvi.01323-24

**Published:** 2025-05-14

**Authors:** Yun-Fa Zhang, Ning Cui, Tong Yang, Jin-Xia Wang, Jia-Hao Chen, Xin Yang, Yong-Xiang Wu, Li-Fen Hu, Xiao-Ai Zhang, Qing-Bin Lu, Xin Su, Hao Li, Wei Liu

**Affiliations:** 1 State Key Laboratory of Pathogen and Biosecurity, Academy of Military Medical Sciences 602528, Beijing, China; 2 The 154th Hospital, China RongTong Medical Healthcare Group Co.Ltd, Xinyang, China; 3 College of Life Science and Technology, Beijing University of Chemical Technology 627792 , Beijing, China; 4 Department of Infectious Diseases, The First Affiliated Hospital of Anhui Medical University 36639, Hefei, Anhui, China; 5 Department of Laboratorial Science and Technology, School of Public Health, Peking University 540412 , Beijing, China; Lerner Research Institute, Cleveland Clinic, Cleveland, Ohio, USA

**Keywords:** SFTS, mtDNA, endothelial cell, B cell, TLR9

## Abstract

Severe fever with thrombocytopenia syndrome (SFTS) is an emerging infectious disease acquired through tick bites. We have previously demonstrated the correlation between SFTSV-induced mitochondrial dysfunction and inflammation induction, disease progression, and fatal outcome. In the current study, our clinical observation study establishes a strong correlation between elevated levels of circulating cell-free mtDNA and poor prognosis. *In vivo* studies further reveal endothelial cells as an important source responsible for releasing mtDNA into circulation, which promotes B cell activation, migration, and differentiation via Toll-like receptor 9 (TLR9). Notably, TLR9 activation enhances B-cell susceptibility to SFTSV infection. These findings suggest that mtDNA released by injured endothelial cells facilitates B cell differentiation and virus replication, emphasizing the significant role of mitochondrial damage within endothelial cells in contributing to the severity of SFTS outcomes.

**IMPORTANCE:**

Severe fever with thrombocytopenia syndrome (SFTS) is a new acute tick-borne infectious disease with a high fatality rate of 10%–50%. There is a strong correlation between SFTSV-induced mitochondrial dysfunction and inflammation induction, disease progression, and fatal outcome. Our research has revealed the crucial role of mtDNA in predicting the prognosis of SFTS and its impact on vascular endothelial injuries as well as B cell differentiation, two previously unexplored features of SFTSV infection. Moreover, mtDNA could activate the TLR9 signal to induce plasmablast differentiation in B cells and promote SFTSV infection. This study provides valuable mechanistic and clinical insights into the adverse outcomes associated with SFTSV infection.

## INTRODUCTION

Severe fever with thrombocytopenia syndrome (SFTS) is a tick-borne disease caused by *Dabie bandavirus*, also known as severe fever with thrombocytopenia syndrome virus (SFTSV). The virus belongs to the genus *Bandavirus* within the family *Phenuiviridae,* order *Bunyavirales* (1, 2). Initially identified in China, SFTS has since been reported in Japan, Korea, Vietnam, Thailand, and other Asian countries (3–6). In recent years, there has been a consistent increase in the number of SFTS cases and an expansion of its geographical distribution, with a mortality rate ranging from 10% to 50% (1, 7–9). In 2017, the World Health Organization (WHO) listed SFTS as one of the top 10 priority diseases (10).

SFTSV infections can present a wide range of clinical spectrums, with most patients experiencing mild illness, whereas some progress to severe diseases and even death (11). Clinical complications associated with SFTS are frequently observed during the second stage of the disease, characterized by neurologic symptoms, hemorrhagic manifestations, and disseminated intravascular coagulation (DIC), accompanied by exacerbated thrombocytopenia and leukopenia, as well as elevated levels of aspartate transaminase (AST), alanine transaminase (ALT), and lactate dehydrogenase (LDH) (12). A significant proportion of severe patients eventually develop multiple organ damage, including acute respiratory distress syndrome (ARDS) and liver dysfunction (13). Notably, plasma leakage has been indicated as a common feature among SFTS patients regardless of disease severity, potentially due to both direct viral infection and indirect stimulation of mast cells, leading to protease secretion and subsequent endothelial cell activation and dysfunction (14, 15). Moreover, the release of damage-associated molecular patterns (DAMPs) by endothelial cells is likely to exacerbate the progression of SFTS (16).

Mitochondria are ubiquitous organelles in eukaryotic cells, characterized by their unique double-membrane structure, and they serve as the core sites for cell energy metabolism (17). These organelles are sensitive to stimuli or external environments, functioning as DAMPs, known as mitochondrial-related DAMPs (mtDAMPs). Upon exposure to cellular oxidative stress, infection, or injury, they trigger subsequent immune responses (18). Studies have demonstrated the crucial role of mitochondria in host immune responses during viral infection and their pivotal involvement in cell death processes (17, 19). In response to viral infection, extracellular/cell-free mtDNA (cf-mtDNA) is released and may accumulate in peripheral blood or tissues, subsequently activating antiviral signaling and inflammatory responses (20). Consequently, an elevated level of cf-mtDNA could potentially serve as a biomarker for diseases or pathological conditions characterized by apoptosis, necrosis, and active cellular secretion (18, 21). Our previous comparative transcriptomic and proteomic analyses of clinical samples and THP-1 cells have revealed a strong correlation between SFTSV-induced mitochondrial dysfunction and the induction of inflammation leading to fatal outcomes (22). However, the underlying mechanisms through which mtDNA acts as a DAMP and contributes to severe disease remain to be elucidated.

Previous studies have shown that cytosine-phosphate-guanine (CpG) motifs within mtDNA could be recognized by various PRRs, including cGAS, TLR9, and inflammasomes. This recognition subsequently triggers the differentiation of plasmablasts, production of cytokines, and expression of costimulatory molecules on B cells (23–25). Conversely, fatal cases of SFTS exhibit a significant expansion of B cells, along with alterations in B-cell subsets, with plasmablasts identified as the primary reservoir for SFTSV replication compared with other B cell subsets (26). All these evidences support our hypothesis that mtDNA might stimulate the TLR9 signaling pathway, thereby promoting plasmablasts differentiation among B cells and facilitating SFTSV infection.

To test this hypothesis, we first examined a cohort of SFTS patients and found significant correlations between elevated levels of circulating cf-mtDNA and increased serum viral loads, as well as unfavorable prognosis. Further *in vitro* assays revealed that mtDNA released from SFTSV-infected endothelial cells can induce B cell activation, migration, and differentiation. Notably, we determined that TLR9 activation affects the susceptibility of B cells to SFTSV infection. These findings enhance our current understanding of the mechanism linking host immune response and adverse outcomes associated with SFTS and provide valuable insights into clinical understanding and therapeutic strategies for managing the disease.

## RESULTS

### High serum cf-mtDNA level is independently associated with fatal outcomes in SFTS patients

Between April 2018 and November 2019, a total of 534 patients were admitted to the 154th hospital with a clinical diagnosis of SFTS. Among them, 23 patients with negative SFTSV RNA and 33 patients with a delay of over 7 days between the disease onset and hospital admission were excluded. Finally, this study enrolled 478 laboratory-confirmed SFTS patients (Fig. S1). These patients had a median age of 64 (IQR, 54–71) years and a male proportion of 41.63% (199/478). Fatal outcomes occurred in 13.39% (64/478) of the patients, with higher fatality rates observed among older individuals (detailed in Table 1).

**TABLE 1:** Epidemiologic and clinical characteristics of SFTS patients^*a*^^,^^*b*^

	All (*N* = 478)	Survival (*N* = 414)	Fatal (*N* = 64)	*P^c^*
Age, median (IQR), years	64 (54–71)	62 (53–70)	72 (66–77)	<0.001
≥65 years, n (%)	225 (47.07)	174 (42.03)	52 (81.25)	<0.001
Male, n (%)	199 (41.63)	172 (41.54)	27 (42.19)	0.923
Interval from onset to admission median (IQR), days	5 (4–6)	5 (4–6)	5 (4–6. 5)	0.049
Length of stay in hospital median (IQR), days	7 (5–9)	8 (6–10)	3 (2-5)	<0.001
Comorbidities, n (%)	182 (38.07)	155 (37.43)	27 (42.19)	0.555
Hypertension	90 (18.82)	78 (18.84)	12 (18.75)	1.000
COPD	53 (11.08)	45 (10.87)	8 (12.50)	0.863
Diabetes	41 (8.58)	34 (8.21)	7 (10.94)	0.628
Hepatitis	29 (6.07)	24 (5.80)	5 (7.81)	0.728
Coronary heart disease	22 (4.60)	19 (4.58)	3 (4.69)	1.000

^*a*^ IQR, interquartile range; COPD, chronic obstructive pulmonary disease.

^*b*^ Bold represents significant statistical differences.

^*c*^ *P*-values were calculated by Mann-Whitney *U* test, or Chi-square test.

**Fig 1:**
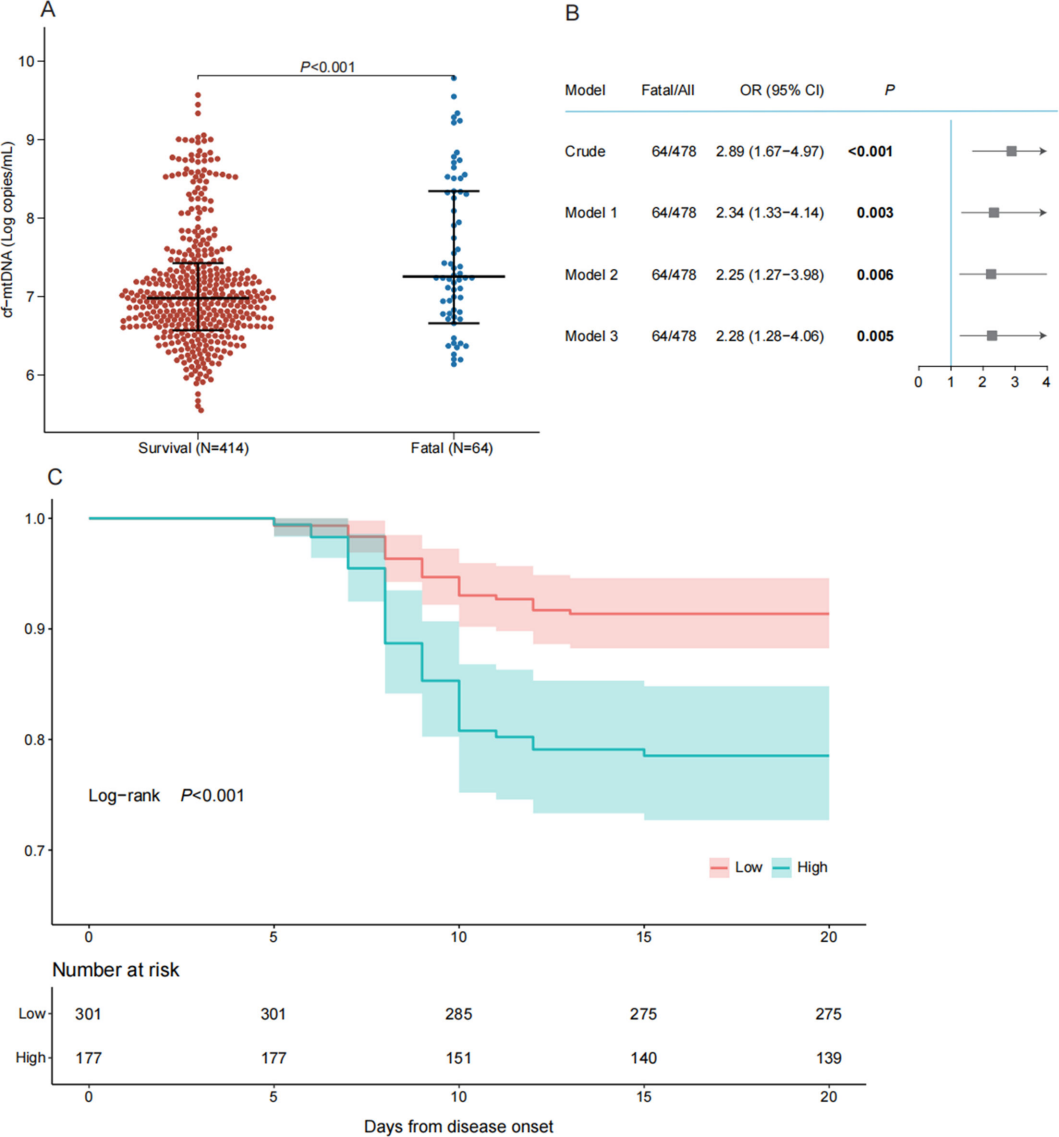
High levels of cf-mtDNA are associated with an elevated risk of death in patients with SFTS. (**A**) Quantification of cf-mtDNA showed a significant difference between survival and fatal groups. (**B**) The odds ratios and 95% CI were estimated from the crude model and three adjusted models. Model 1 adjusted for age; Model 2 adjusted for age and sex; and Model 3 adjusted for age, sex, and the presence of comorbidities. (**C**) Kaplan-Meier curves on the probability of survival in two groups with high and low cf-mtDNA levels. The numbers of at-risk patients at each time point were shown for each group. *P* values were calculated by log-rank test.

The levels of cf-mtDNA were quantified in samples collected upon admission to the hospital from all 478 enrolled SFTS patients. Significantly higher levels of cf-mtDNA were observed in the 64 fatal SFTS patients [7.25 (6.79–8.34)] compared with the 414 survivors [6.98 (6.63–7.36), *P* < 0.001] (Fig. 1A). In three multivariable logistic regression models that included different variables for adjustment, a high copy number of cf-mtDNA remained significantly associated with an increased risk of death, with adjusted odds ratios (ORs) ranging from 2.25 to 2.34 (Fig. 1B). When categorizing cf-mtDNA based on a cutoff value of 7.21 log copies/mL, it was found that patients in the high-cf-mtDNA group had a significantly higher risk of developing a fatal outcome, compared with those in the low-cf-mtDNA group, as indicated by Kaplan-Meier curves and log-rank tests (*P* < 0.001) (Fig. 1C). These findings suggest that serum cf-mtDNA levels could serve as an early biomarker for predicting fatal outcomes in SFTS patients.

**Fig 2:**
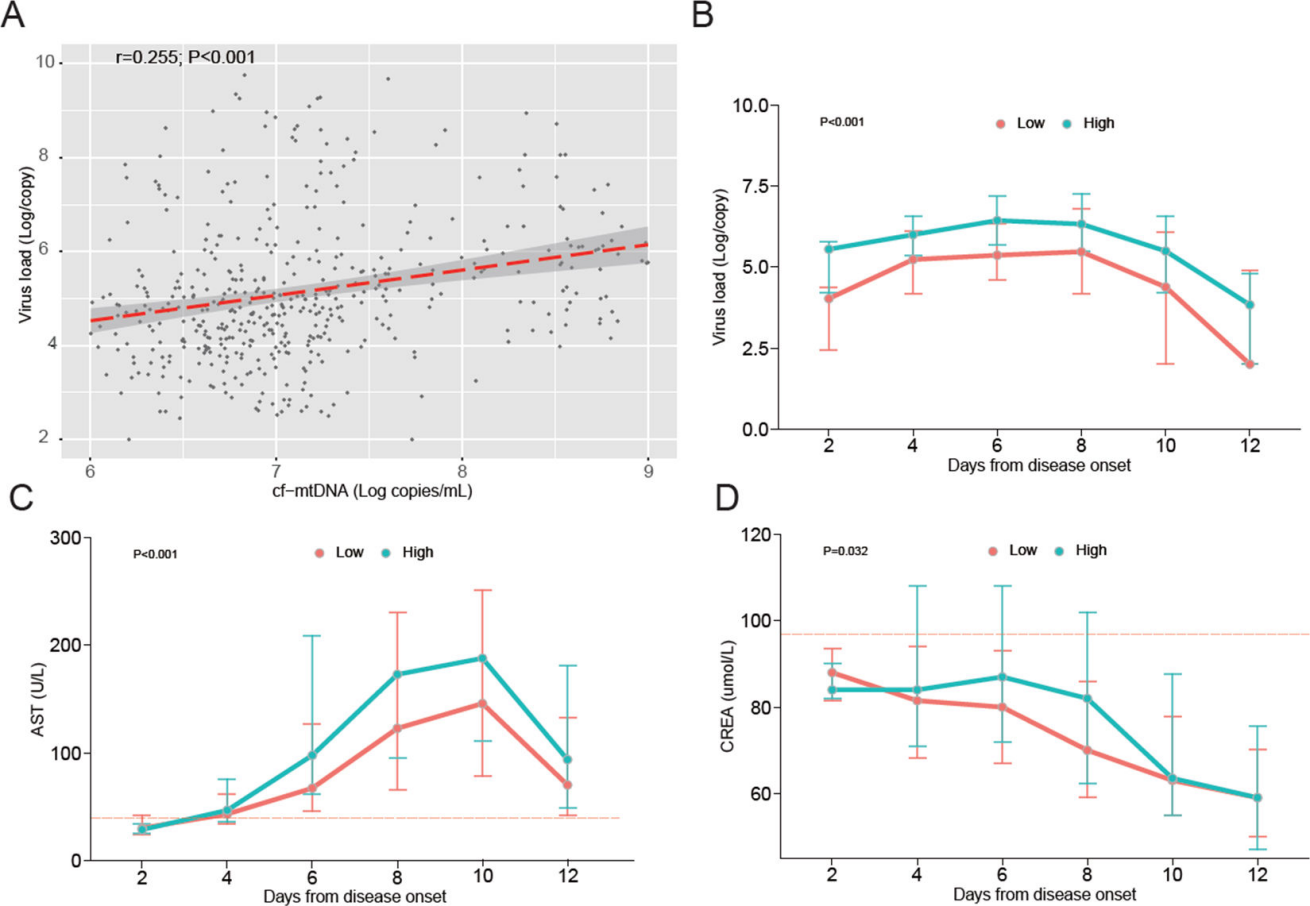
Measurement of cf-mtDNA in relation to virus load (A and B), AST (**C**), and CREA (**D**) in SFTS patients. (**A**) Spearman’s correlation coefficient was used to assess the correlation between cf-mtDNA copy number and viral loads. The red line represents the fitted regression line, and the gray area represents the 95% CI. (B–D) Kinetics of viral loads (**B**), AST (**C**), and CREA (**D**) compared between high and low cf-mtDNA groups with SFTSV infection. Data points are median values, and error bars show interquartile ranges (IQR). *P* values were calculated by the GEE model. The short red-dotted line represents the upper limit of the normal value of the indicator.

We further demonstrated a positive correlation between cf-mtDNA copy number and viremia levels, which were evaluated using the same sample (r = 0.255, 95% CI: 0.156–0.394, *P* < 0.001, Fig. 2A). Utilizing a generalized estimating equation (GEE) model to compare dynamic changes in repeatedly measured viral loads and laboratory indicators between the two groups, we observed significantly higher levels of viremia, AST, and creatinine (CREA) in the high cf-mtDNA copy group compared with the low cf-mtDNA copy group (all *P* < 0.05) (Fig. 2B through D).

### SFTSV infection causes mitochondria dysfunction in endothelial cells

**Fig 3:**
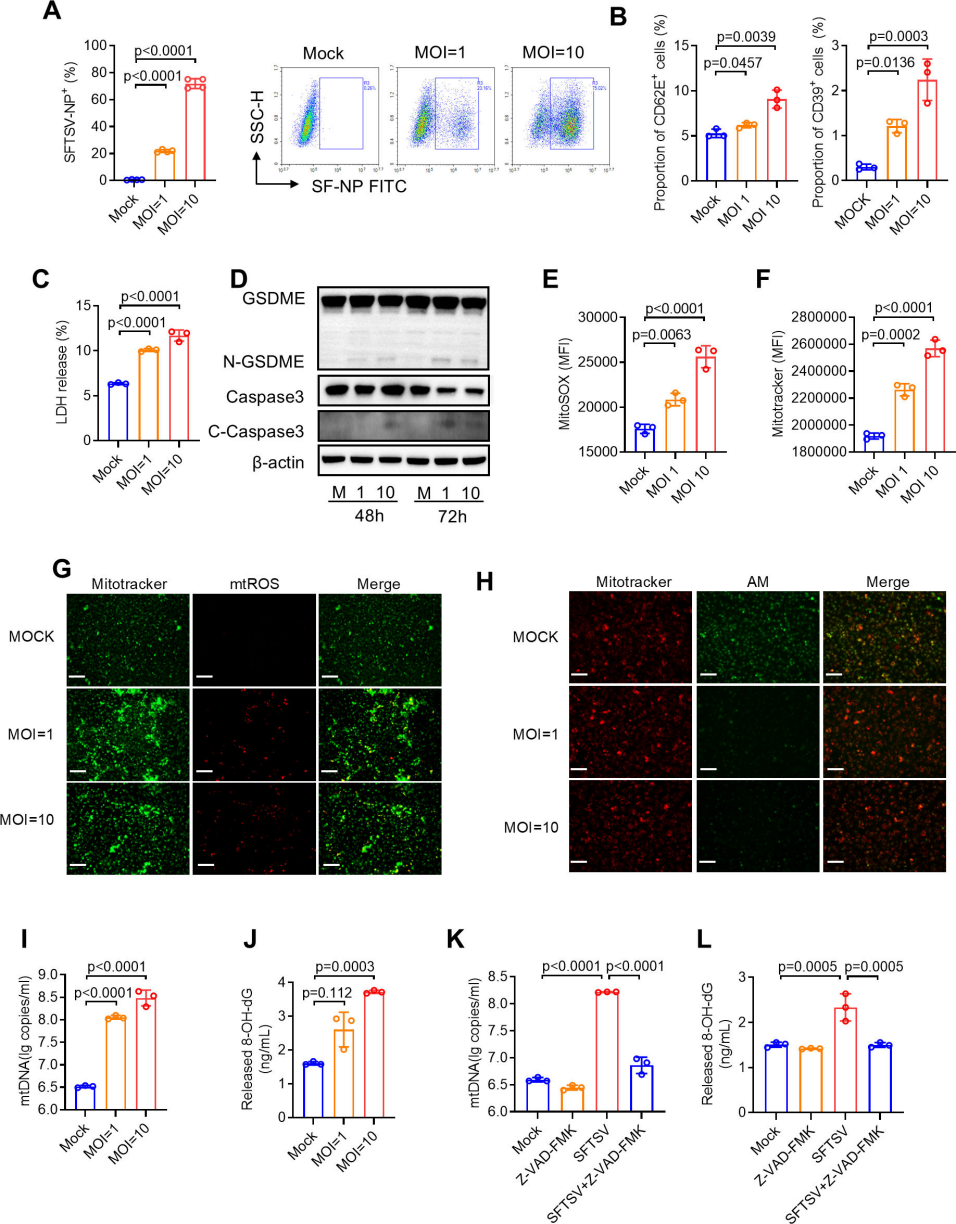
SFTSV induces endothelial cell damage and mitochondrial DNA release in HUVECs. (**A**) Flow cytometry analysis of SFTSV infection rate in HUVECs at 72 h post-infection (hpi). (**B**) The proportion of CD62E^+^ (left panel) and CD39^+^ (right panel) cells measured by flow cytometry in SFTSV-infected HUVECs at 72 hpi. (**C**) LDH release examined by the CytoTox 96 Non-Radioactive Cytotoxicity Assay Kit in SFTSV-infected HUVECs at 72 hpi. (**D**) Representative western blot images of HUVECs lysates post-SFTSV infection, processed for pyroptotic protein levels using GSDME, Caspase3, and C-Caspase3 specific antibody. β-Actin protein was tested as an internal loading control. (E and F) HUVECs were subject to flow cytometric analysis of mitochondrial reactive oxygen species (mtROS, **E**) and mitochondria mass (MitoTracker, **F**) at 72 hpi. (**G**) Immunofluorescence analysis of MitoTracker (Green) and mtROS (Red) in SFTSV-infected HUVECs at 72 hpi. Scale bar: 100 µm, Images shown are representative of three independent experiments. (**H**) HUVECs were infected with SFTSV for 72 h; then, immunofluorescence analysis of MitoTracker (Red) and AM (Green) in SFTSV-infected HUVECs at 72 hpi was performed to evaluate the opening of mitochondrial permeability. (**I**) The extracellular mtDNA released from HUVECs was detected by qPCR at 48 hpi. (**J**) The release of 8-OH-dG in the supernatant of HUVECs at indicated MOIs for 72 h. (K and L) HUVECs were infected with SFTSV and treated with Z-VAD-FMK for 72 h. The extracellular mtDNA (**K**) and 8-OH-dG (**L**) were analyzed. Data are present as mean ± s.d.

Given that vascular injuries and endothelial dysfunction are features of SFTS (27), we aimed to investigate whether SFTSV infection independently triggers mitochondrial dysfunction and the release of extracellular mtDNA in vascular endothelial cells. Initially, we determined that human umbilical vein endothelial cells (HUVECs), a type of endothelial cell, exhibited high susceptibility to SFTSV infection, with approximately 70% of the cells testing positive for SFTSV NP^+^ (Fig. 3A). Furthermore, flow cytometry analysis revealed a significant upregulation of CD62E (an indicator of endothelial cell activation) and CD39 (an indicator of endothelial cell inflammation) in an MOI-dependent manner at 72 hpi (Fig. 3B). Upon infection with SFTSV at an MOI of 5 or 10, HUVECs culture displayed an elevated LDH release compared with non-infected controls, suggesting that endothelial cell injury causes SFTSV infection (Fig. 3C). Further western blot analysis revealed a significant increase in the protein levels of cleaved caspase-3 and activated GSDME (N-GSDME) during SFTSV infection (Fig. 3D), indicating that SFTSV-induced endothelial injury may be associated with pyroptosis.

In addition, mitochondrial ROS also significantly accumulated with SFTSV infection, accompanied by an increase in mitochondrial mass (Fig. 3E through G). Following SFTSV infection, an MOI-dependent downregulation of calcein acetoxymethyl ester (Calcein AM) signal located in mitochondria was observed, indicating the opening of mitochondrial permeability transition pore (mPTP) (Fig. 3H). Additionally, SFTSV infection resulted in an MOI-dependent increase in mtDNA content released into the culture medium of HUVECs (Fig. 3I). Similar results have been reported in previous studies, which showed that the accumulation of mtROS induces mitochondrial stress, leading to the opening of mPTP and subsequent leakage of mitochondrial contents into the cytoplasm (28, 29). Previous studies have shown that SFTSV infection induced mtROS production, leading to mtDNA oxidation (22). Our study further revealed that the concentration of 8-OH-dG, an indicator of DNA oxidation, progressively increased in the supernatant as the MOI rose (Fig. 3J). This suggests that mtDNA could be oxidized during SFTSV infection. Moreover, the release of mtDNA is associated with pyroptosis (30, 31). We observed that the accumulation of mtDNA and 8-OH-dG in the supernatant was suppressed following the inhibition of GSDME-mediated pyroptosis using Z-VAD-FMK, a pan-caspase inhibitor (Fig. 3K and L). The fact that SFTSV induces BAK/BAX-dependent release of mitochondrial DNA, as previously documented (22), corroborates the current finding that SFTSV infection directly triggers mitochondrial dysfunction and pyroptosis, leading to extracellular release of mtDNA in HUVECs.

### The released mtDNA induces B cell activation, migration, and differentiation

**Fig 4:**
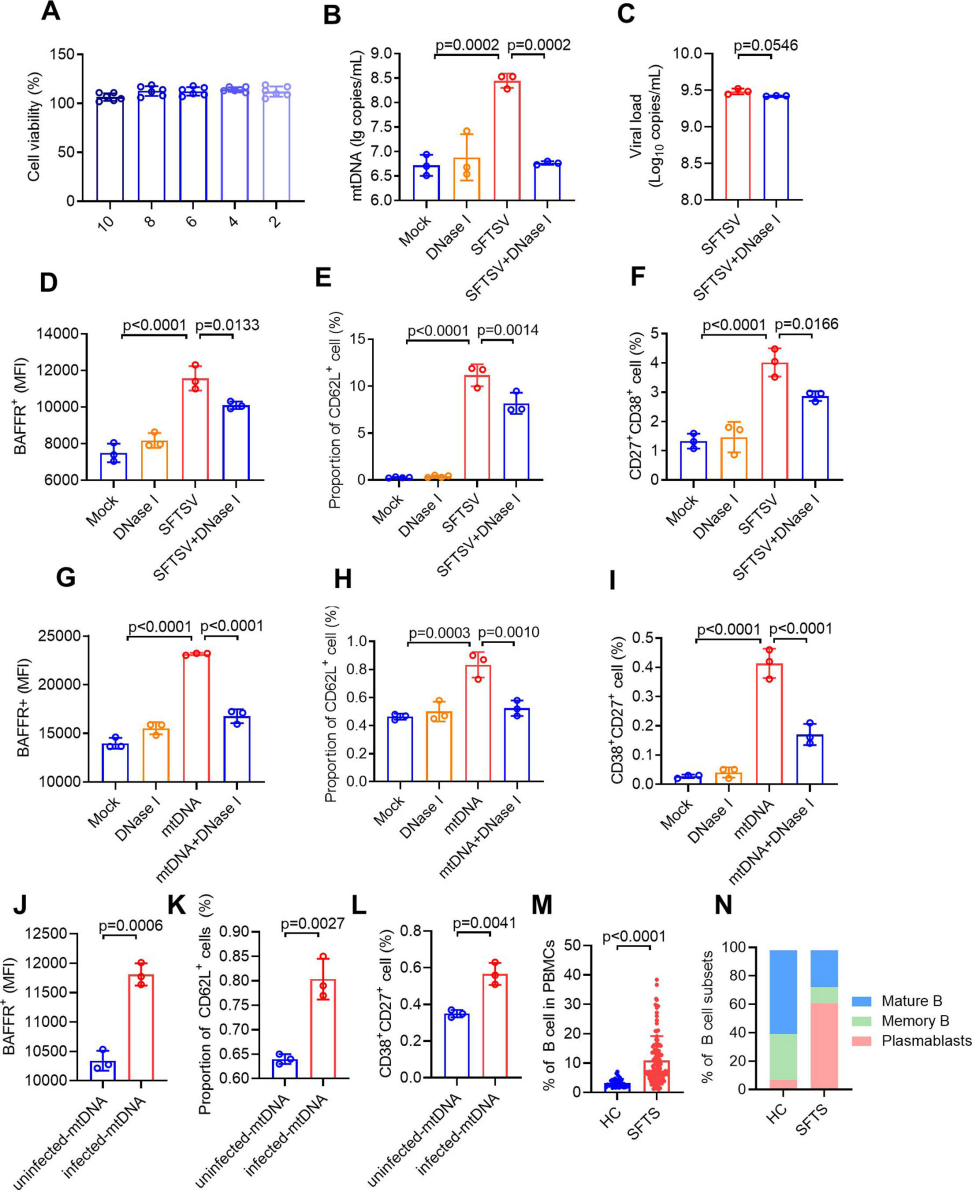
HUVECs release mtDNA that induces B cell activation and differentiation. (A) Viability of DNase I-treated HUVECs was measured using CCK-8 at 72 h post-infection (hpi). (B and C) SFTSV-infected HUVECs were treated with DNase I (10 U/mL) for 72 h, and mtDNA levels (B) and viral load (C) in the supernatant were measured with RT-qPCR. (D–F) HUVECs were infected with SFTSV at MOI = 5 or treated with DNase I (10 U/mL) for 24 h, followed by co-culture with Nalm-6 cells for 72 h. The proportion of BAFFR^+^ (D), CD62L^+^ (E), and plasmablasts (CD27^+^ CD38^+^ cells) (F) in Nalm-6 cells was detected by flow cytometry at 72 h post-co-culture. (G–I) B cells were isolated from PBMCs and treated with mtDNA purified from SFTSV-infected HUVECs (MOI = 5) rather than the cell culture supernatant or treated with DNase I (10 U/mL). The proportion of BAFFR^+^ (G), CD62L^+^ (H), and plasmablasts (CD27^+^ CD38^+^ cells) (I) was detected by flow cytometry at 72 h. (J–L) Nalm-6 cells were treated with mtDNA purified from SFTSV-infected HUVECs or non-infected HUVECs. The proportion of BAFFR^+^ (J), CD62L^+^ (K), and plasmablasts (CD27^+^ CD38^+^ cells) (L) was detected using flow cytometry at 24 h. (M) The proportion of B cells in PBMCs from healthy controls (HC) and SFTS patients was measured using flow cytometry. (N) Subpopulation of B cells in PBMCs from HC group and SFTS patients. Data represent the mean ± s.d.

To investigate the impact of mtDNA on B cell activation and differentiation, we established a co-culture model in which HUVECs were infected with SFTSV for 24 h, followed by a 72 h co-cultured with B cells after removing the culture supernatant. As a negative control, DNase I treatment was employed to degrade the mtDNA in the culture supernatant without causing cytotoxic or antiviral effects (Fig. 4A through C). BAFFR, a survival receptor that regulates B-cell maturation, serves as an indicator of B cell activation (32). By using BAFFR^+^ staining, we observed a significant upregulation of BAFFR following SFTSV infection, whereas no such increase was seen in the DNase I treatment group (Fig. 4D). CD62L (L-selectin), an adhesion molecule involved in lymphocyte adherence to endothelial cells in peripheral lymph nodes (33), was also significantly upregulated on B cells after SFTSV infection. This upregulation was inhibited when mtDNA was degraded by DNase I treatment (Fig. 4E). Flow cytometry analysis revealed a significant expansion of plasmablasts (CD27^+^CD38^+^) following SFTSV infection, but no significant differences were observed in the DNase I treatment group (Fig. 4F). Furthermore, when mtDNA purified from SFTSV-infected HUVECs was used to treat B cells, we observed an induction of activation, migration, and differentiation of B cells compared with non-mtDNA-treated controls; however, these effects were suppressed by DNase I treatment (Fig. 4G through I). In addition, the mtDNA purified from non-infected HUVECs exhibited a modest capacity to stimulate Nalm-6 cells (Fig. S2A through C). However, the mtDNA purified from SFTSV-infected HUVECs was more effective for B cell stimulation than the mtDNA from non-infected HUVECs, including the upregulation of BAFFR, CD62L, and CD27^+^CD38^+^(Fig. 4J through L). Interestingly, an expansion of both B cells and plasmablasts was also identified in SFTS patients compared with healthy controls (Fig. 4M and N). These findings collectively indicate that mtDNA released from SFTSV-infected HUVECs subsequently promotes B cell activation, migration, and differentiation.

### mtDNA enhances the susceptibility of B cells to SFTSV

**Fig 5:**
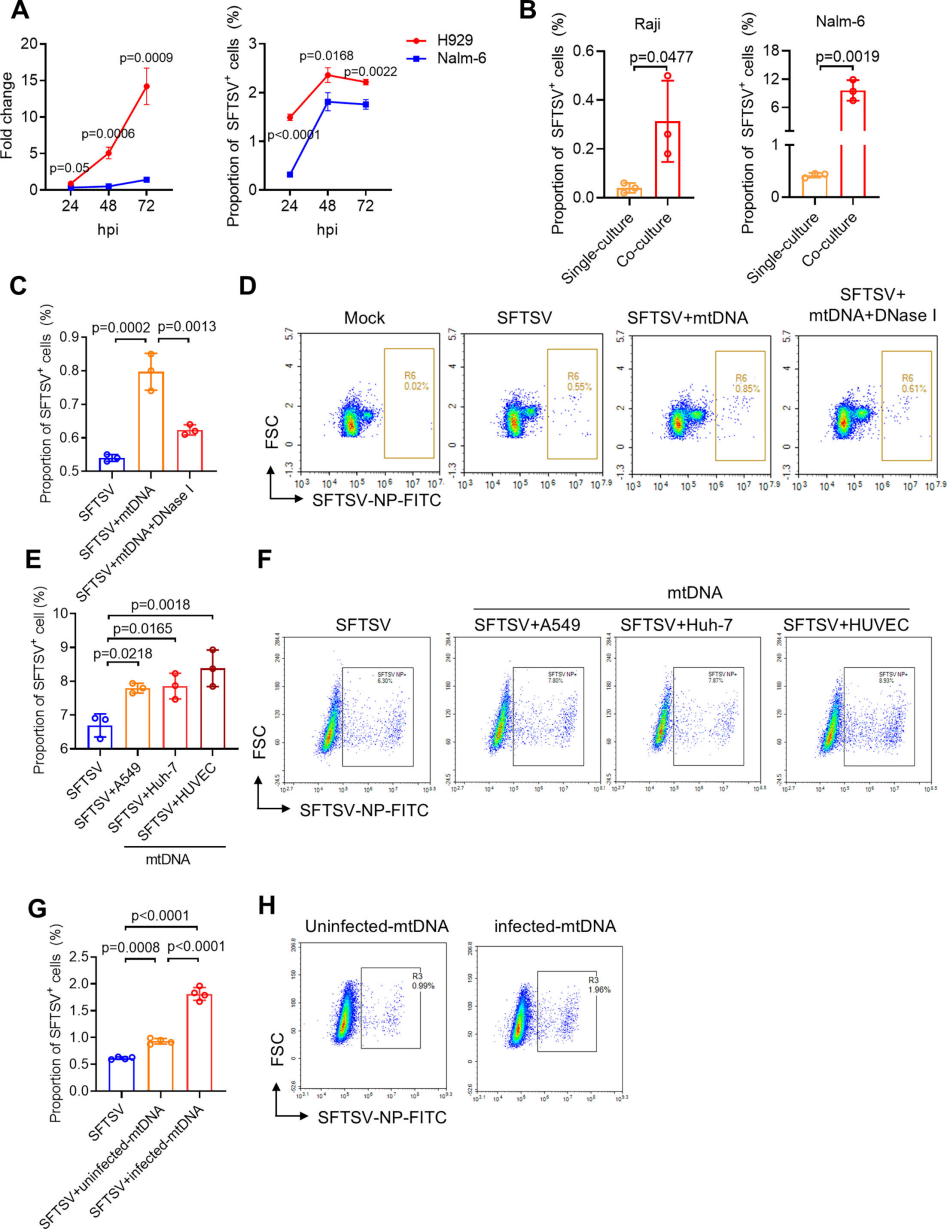
HUVCE-derived mtDNA promotes the susceptibility of B cells to SFTSV. (**A**) The relative levels and proportion of SFTSV NP^+^ cells in plasmablasts cell lines (H929) and naive B cell lines (Nalm-6) at 72 h post-infection with SFTSV (MOI = 5). (**B**) The proportion of SFTSV NP^+^ cells in two B cell lines (Raji and Nalm-6) infected with SFTSV (MOI = 5) (single-culture group), or additionally co-cultured with HUVECs (co-culture group) at 72 h post-infection. (C and D) Effect of mtDNA, purified from SFTSV-infected HUVECs rather than the cell culture supernatant, treatment on the proportion of SFTSV NP^+^ cells in primary B cells isolated from human peripheral blood at 72 h post-infection (**C**). Representative images of flow cytometry analysis were shown (**D**). (E and F) Effect of different types of mtDNA, purified from SFTSV-infected A549, Huh-7, and HUVEC, treatment on the proportion of SFTSV NP+ cells in Nalm-6 (**E**), representative images of flow cytometry analysis were shown (**F**). (G and H) Effect of mtDNA from infected or non-infected HUVECs on the susceptibility of Nalm-6 to SFTSV (**G**). Representative images of flow cytometry analysis were shown (**H**). Data presented as mean ± s.d.

We have demonstrated that SFTSV infection induces mtDNA release from HUVECs, promoting B cells differentiation into plasmablasts. Previous studies have associated robust B-cell expansion and immune activation with fatal outcomes in SFTSV infection (34). Therefore, we further investigated whether mtDNA modulates the susceptibility of B cells to SFTSV. Our findings revealed a significantly higher susceptibility and replication efficiency of SFTSV in the plasmablast cell line (H929) (35), compared with the naive B cell line (Nalm-6) (36) (Fig. 5A). Moreover, co-culturing B lymphocyte cell line (Raji) with Nalm-6 cell line resulted in a significantly increased proportion of SFTSV NP^+^ positive population compared with single-culture conditions (Fig. 5B). Furthermore, flow cytometry analysis showed that treatment with mtDNA elevated the proportion of SFTSV NP^+^ population in primary B cells, which was reduced upon degradation of mtDNA by DNase I (Fig. 5C and D). Similar to mtDNA derived from HUVECs, another type of purified mtDNA from SFTSV-infected A549 or Huh-7 also increased susceptibility to SFTSV (Fig. 5E and F). Similar to the effect of stimulating B cells, SFTSV-infected mtDNA enhanced B cell susceptibility to SFTSV more effectively compared with the mtDNA from non-infected HUVECs (Fig. 5G and H), which might be associated with oxidized mtDNA. Overall, these results suggest that mtDNA may enhance the susceptibility of B cells to SFTSV by stimulating their differentiation from naïve B cells to plasmablasts.

### TLR9 signaling is involved in B cell activation, migration, differentiation, and susceptibility to SFTSV

**Fig 6:**
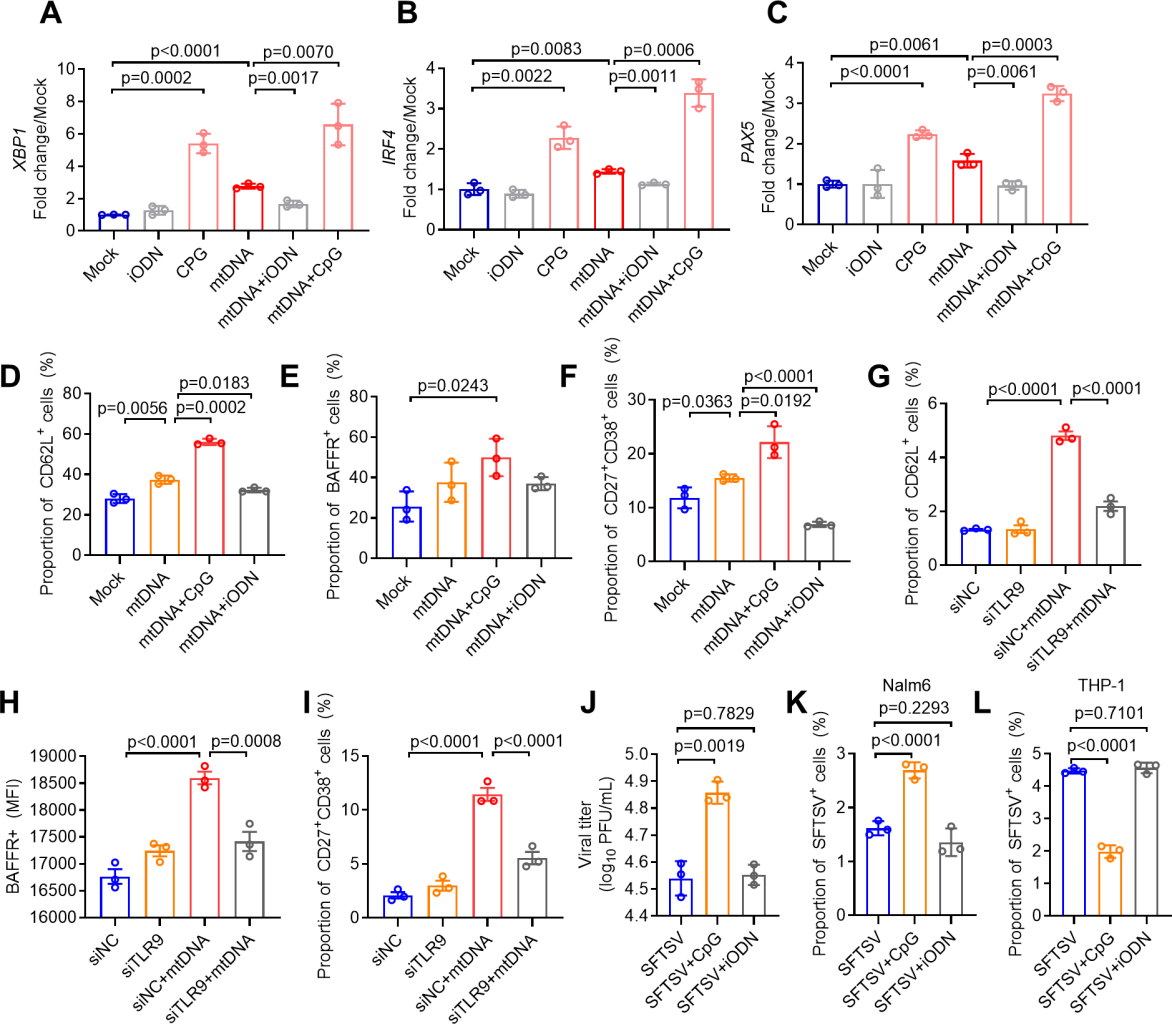
HUVEC-released mtDNA induces B cell activation and differentiation through the TLR9-signaling pathway. (A–C) B cells isolated from peripheral blood treated with 6 µg/mL CpG (a TLR9 agonist) and 0.5 µM iODN (a TLR9 inhibitor) were incubated with mtDNA for 72 h. The expression of differentiation-related genes *XBP1* (**A**), *IRF*4 (**B**), and *PAX5* (**C**) in B cells was measured with quantitative RT-PCR (qRT-PCR). (D–F) The proportion of CD62L^+^ (**D**), BAFFR^+^ (**E**), CD27 (F), and^+^ CD38^+^ (**F**) among B cells. (G–I) TLR9-knockdown Nalm-6 cells were treated with mtDNA purified from SFTSV-infected HUVECs. The proportion of CD62L^+^ (**G**), BAFFR^+^ (**H**), and CD27^+^ CD38^+^ (**I**) was examined. (**J**) SFTSV-infected B cells treated with CpG and iODN. The supernatant viral titer was measured with the immunological focus assay. (K and L) Effect of CpG (6 µg/mL) and iODN (0.5 µM) on the proportion of SFTSV NP^+^ cells in Nalm-6 (**K**) and THP-1 (**L**). Data presented as the mean ± s.d.

The previous report indicated that mtDNA treatment could stimulate an immune response in neutrophils via the TLR9 signaling pathway (37). To investigate the effect of TLR9 activation on B cells, human primary B cells were treated with CpG (a TLR9 agonist) at a concentration of 6 µg/mL and iODN (a TLR9 inhibitor) at a concentration of 0.5 µM. Both CpG and mtDNA treatments upregulated the expression of genes associated with B cell differentiation, including *XBP1, IRF4,* and *PAX5*; however, this enhancement was reduced by iODN treatment (Fig. 6A through C). Additionally, CpG synergized with mtDNA to facilitate the expression of CD62L, BAFFR, and CD27^+^CD38^+^ of B cells, which were significantly blocked by iODN treatment (Fig. 6D through F). Similarly, knockdown of TLR9 restrained the activation, migration, and differentiation of B cells by mtDNA treatment, suggesting an important role of TLR9 signaling in mtDNA-triggered activation, differentiation, and migration of B cells during SFTSV infection (Fig. 6G through I). Moreover, CpG treatment increased the susceptibility of B cells to SFTSV infection, which was not observed for iODN treatment (Fig. 6J and K). To determine whether TLR9 is necessary for SFTSV infection, we used the human monocytic cell line THP-1 as a negative control, since it expresses TLR9 and is susceptible to SFTSV. Activation of TLR9 with CpG treatment reduced the susceptibility of THP-1 to SFTSV infection (Fig. 6L). In addition, treatment with mtDNA purified from SFTSV-infected HUVECs resulted in a decreased infection rate of THP-1 and 293T cells, indicating that the effect of mtDNA on enhancing susceptibility to SFTSV might be specific to B cells (Fig. S3A through D). Consistent with the previous study, TLR9 signaling inhibited SFTSV replication in HEK 293T cells (38). Therefore, TLR9 signaling is not a prerequisite for SFTSV infection. These findings support the role of TLR9 activation in promoting B cell activation, migration, differentiation, and increasing susceptibility to SFTSV.

## DISCUSSION

Recent investigations have determined various biomarkers associated with the severity of SFTS. These include elevated serum levels of AST/ALT, LDH, BUN, etc., indicating damage to the liver, kidney, and other organs (39, 40). In our study, we determined a significant association between early infection-induced increase in cf-mtDNA levels and an elevated risk of mortality. This finding correlated with higher viremia and more severe organ dysfunction. A similar phenotype characterized by significantly higher levels of cf-mtDNA in plasma has been reported in cases involving severe sepsis and multiple infectious diseases (41, 42). Enhanced mitochondrial dysfunction was observed in HUVECs infected with SARS-CoV-2, characterized by increased production of mitochondria-derived superoxide anion, elevation of mitochondrial membrane potential, and release of mtDNA (43). Moreover, similar to the previously established predictive capacity of plasma cf-mtDNA for disease outcomes (44, 45), our findings now uncovered the ability of mtDNA to predict disease severity during SFTSV infection, which reflects the ongoing process of cell death. This represents robust evidence based on a large clinical cohort. However, there are current challenges in utilizing mtDNA as a clinical diagnostic tool and prognostic biomarker. The range of mtDNA levels caused by SFTSV infection overlapped between deceased and surviving patients, hindering its effective application in prognostic diagnosis. This phenomenon is also observed in other studies, such as human immunodeficiency virus and chronic graft-versus-host disease (41, 46). Drawing from the research on mtDNA in SARS-CoV-2 (47), we propose that a comprehensive analysis incorporating mtDNA alongside other biomarkers, such as LDH, ferritin, and IL-6, could assist in predicting the clinical prognosis of SFTS (48). Continuous monitoring of individual patients' mtDNA levels throughout the disease course is also recommended.

Despite a previously noted association between mtDNA and poor outcomes in SFTS cases, the underlying mechanisms remain poorly examined. Our previous study provides crucial insights, revealing that SFTSV-induced mtDNA triggers NLRP3 inflammasome activation, thereby promoting the development of severe SFTS (22). Herein, we further demonstrated that endothelial cells are a source responsible for releasing mtDNA into circulation. Given that our previous study has determined vascular endothelial cell injury and barrier function damage as key pathogenesis processes following SFTSV infection, leading to subsequent pathophysiological effects (14, 27), this evidence collectively could help elucidate the mechanism by which mtDNA mediates the pathogenic process during human SFTSV infections and contributes to fatal outcomes. Mitochondrial damage is regulated by multi-factors, all of which may lead to mtDNA release (49). However, our previous studies indicate that the majority of mitochondrial proteins are downregulated during SFTSV infection, and only the mitochondrial protein BCL2 antagonist/killer (BAK) is significantly upregulated. These phenomena further hint at the possibility that SFTSV induces mtDNA release in endothelial cells via BAK/BAX (22). In addition, endothelial cells also play a major role in recruiting neutrophils to expand the inflammatory site and regulate inflammatory reactions. This process may involve DAMPs represented by mtDNA (50, 51). For example, circulating mtDNA binding to neutrophils triggers net formation, which may play a central role in multiple organ failure and inflammatory response (52).

Recently, it has been noted that early infection of SFTSV triggers a robust IFN-I response in B cells, particularly plasmablasts, which contributes to poor outcomes at the terminal stage of the disease (53). Clinical data from deceased SFTS patients also revealed impaired serological responses to SFTSV, including a deficiency in specific IgG antibodies targeting viral nucleocapsid and glycoprotein, attributed to defective B-cell class switching (54). Considering the known pathogenesis of SFTS and the potential role of mtDNA as an endogenous ligand and TLR9 stimulus, we further demonstrated that the mtDNA-TLR9 pathway is involved in exacerbating B cell differentiation into plasmablasts and promoting SFTSV infection. Furthermore, previous studies have indicated that SFTSV infection-induced mtROS plays a role in oxidizing mtDNA, which in turn potentiates TLR9 activation (22, 55). We also observed an increase in both mtDNA and 8-OH-dG in HUVECs (Fig. 3I and J). These findings collectively suggest that the pronounced influence of mtDNA from SFTSV-infected HUVECs on B cell differentiation and susceptibility compared with normal mtDNA may be due to the oxidation of mtDNA, which may exhibit a higher capacity to promote the expression of TLR9 and induce immune response compared with non-oxidatively modified mtDNA (55, 56). Additionally, although previous studies have shown that oxidation can lead to mutations in mtDNA sequences (57), we observed no significant mutation of mtDNA caused by SFTSV infection (Fig. S4). This may represent a strategy evolved by SFTSV to exploit TLR9 for mediating B-cell responses in favor of self-replication (58). These current findings suggest that restoring mitochondrial homeostasis or inhibiting TLR9 signaling could serve as potential targets for developing non-specific therapeutic strategies against viral infections. However, recent studies have shown that under optimal stimulation conditions using the TLR9 ligand CpG-A, B cells were capable of producing type I IFN *in vivo* (59), indicating the involvement of TLR9 in antiviral processes. Therefore, additional *in vivo* and clinical data are needed to support the possibility of TLR9-based therapy for treating SFTS.

There are several limitations in the present study. Notably, the TLR9 signaling cascade culminates in the activation of the transcription factor nuclear factor kappaB (NF-κB), which plays a role in facilitating the development and survival of B cells (60), as well as the production of natural IgM antibodies (61). These observations suggest the potential significance of the TLR9/NF-kB pathway in modulating B cell activation during SFTSV infection. Nevertheless, detailed molecular mechanisms by which TLR9 affects B cell activation and differentiation during viral infection need further exploration.

Taken together, our findings from both clinical observation and *in vivo* co-culture model provide compelling evidence for the significant role of cf-mtDNA in predicting the prognosis of SFTS and B cell differentiation, which are less explored aspects of SFTSV infection. Moreover, we have demonstrated that mtDNA activates the TLR9 signaling pathway, resulting in B cell differentiation into plasmablasts and facilitating SFTSV infection. This study provides valuable mechanistic insights and clinical implications regarding adverse outcomes associated with SFTSV infection. Future studies on the interaction between mtDNA and other immune cells are warranted to elucidate the pathophysiological effects caused by mtDNA.

## MATERIALS AND METHODS

### Study design, participants, and data collection

A retrospective cohort study was performed at the 154th Hospital in Xinyang, Henan province in China from 2018 to 2019. Patients who tested positive for SFTSV RNA by real-time RT-PCR were defined as laboratory-confirmed cases of SFTS and included in this study. Patients with delayed hospitalization admission (>7 days from disease onset to hospital admission) or a short hospitalization duration (< 3 days from disease onset to hospital discharge or death) were excluded (Fig. S1). Baseline demographics, medical history, and clinical symptoms/syndromes were collected and recorded in the EpiData database. Routine laboratory measurements were extracted from the medical records of the hospital. The primary outcome was survival or death, determined through the medical records or follow-up of patients who discontinued therapy or were discharged due to adverse clinical progression or economic reasons within 1 month after discharge.

### Human blood collection and serum preparation

Blood was collected within 24 h of hospitalization using pro-coagulation BD vacutainer tubes. The blood samples were then centrifuged to obtain serum. After purification through a 0.45 µm microfiltration membrane to remove platelets or cell debris, purified serum was obtained and stored in sterile nuclease-free tubes at −80°C for later analysis. Heparinized peripheral blood was collected from healthy volunteers of comparable age as the patients after obtaining written informed consent from all the subjects and approval from the ethics committee of the Academy of Military Medical Sciences.

### Cells and viruses

HUVECs, obtained from the American Type Culture Collection (ATCC, USA), were maintained in Endothelial Cells Medium (ScienCell, USA) supplemented with 5% fetal bovine serum and 1% endothelial cell growth factor (ECGF). Vero cells obtained from ATCC were maintained in Dulbecco’s modified Eagle’s medium. B cell lines (Nalm-6, Raji, and H929 cells) obtained from ATCC were cultured in RPMI-1640 medium containing 10% FBS and 1% penicillin-streptomycin solution. All cells were cultured at 37°C in a humidified atmosphere with 5% CO_2_.

The SFTSV strain HBMC16 (GenBank: KY440775.1, KY440776.1, and KY440777.1), isolated by the Wuhan Institute of Virology, Chinese Academy of Sciences (Wuhan, Hubei, China), was propagated in Vero cells for use in this study. Infection was conducted by incubating SFTSV on monolayer Vero cells for 3 days at 37°C in a humidified atmosphere with 5% CO_2_, and then, the cell culture supernatants were harvested. Viral titer was determined by focus-forming assay on Vero cells using a two-step immunostaining method with an antibody against viral protein NP and an anti-mouse horseradish peroxidase-conjugated secondary antibody.

### Human primary cells

Peripheral blood mononuclear cells (PBMCs) were obtained from the heparinized peripheral blood through density gradient centrifugation using Ficoll-Paque Plus (GE, 17144002). The PBMC fractions were treated with RBC Lysis Buffer (BioLegend, 420301) to remove red blood cells and washed with Ca/Mg-free PBS. For the isolation of B lymphocytes, PBMCs were further separated using the EasySep Direct Human Total Lymphocyte Isolation Kit (Stemcell Technologies, 17954) according to the manufacturer’s instructions. After isolation, PBMCs/B cells were cultured in RPMI 1640 medium supplemented with 10% heat-inactivated FBS at 37°C. All sample preparation was done at room temperature.

### mtDNA isolation

Mitochondrial DNA was isolated using a Mitochondria DNA Isolation kit (Abcam, ab65321). Briefly, cell samples rather than supernatant were collected using 1 mL of 1× Cytosol buffer and then incubated on ice for 10 min. The cells were homogenized using a Dounce tissue grinder to ensure that 70%–80% of the cell membranes were ruptured. Gradual removal of cellular debris was achieved by adjusting the rotational speed to obtain a mitochondrial precipitate, which was then lysed using 30 µL of mitochondrial lysis buffer. Finally, ethanol precipitation was employed to isolate the mitochondrial DNA after protein degradation. To determine the effect of mtDNA, DNase I was used to degrade mtDNA as described in a previous study (62). In general, the mtDNA content was determined using qPCR after the DNase I (5 U/mL) and SFTSV dealing with cells at 72 hpi.

### Quantification of mtDNA

For mtDNA from the serum of SFTS patients, which was extracted using QIAmp DNA Blood Mini Kit (Qiagen, USA). For the detection of mtDNA in the supernatant, DNA was extracted from the supernatant using the TIANamp virus DNA/RNA kit (TIANGEN, DP315). The mtDNA was detected by qPCR assays by using *Premix Ex Taq* (Probe qPCR) (Takara, RR390A) on the LightCycler 480 II real-time PCR platform (Roche, Swiss). Furthermore, the concentration of mtDNA was assessed using primers targeting mitochondrially encoded NADH dehydrogenase 1 (63). The primers for real-time PCR were designed using Primer-BLAST from the National Center for Biotechnology Information (USA) and obtained from Eurogentec (Belgium). All clinical samples used for mtDNA testing have been ensured to be free from bacterial or endotoxin contamination.

### Measurement of mtROS mitochondrial mass and mitochondrial permeability

HUVECs were either mock-treated or infected with SFTSV. They were then stained with MitoSOXTM Red mitochondrial superoxide indicator (Invitrogen, M36008), Mito-Tracker Red CMXRos (Beyotime C1049B-50 μg), or MitoTracker Green FM for 30 min at 37°C in darkness according to manufacturer’s instructions. For MitoProbe Transition Pore (mPTP, Invitrogen, cat. M34153), each cell sample was divided into two tubes: one tube contained calcein AM and CoCl_2_ for mitochondrial calcein staining alone, whereas the other tube contained calcein AM, CoCl,_2_ and ionomycin. The difference in fluorescence intensity between the two tubes reflects the extent of mPTP opening. All indicators were analyzed using flow cytometry and observed under an epifluorescence microscope (DMi 8, Leica).

### Measurement of cell death by LDH release

The release of LDH in the supernatant from cell culture was examined by using CytoTox 96 Non-Radioactive Cytotoxicity Assay Kit (Promega, G1780) according to the manufacturer’s protocol. Briefly, 50 µL supernatant from cell culture was mixed with 50 µL CytoTox 96 Reagent and incubated at room temperature for 15 min. The reaction was terminated by adding a stop solution, and the absorbance at 490 nm was determined for each well using a microplate reader.

### Measurement of 8-OH-dG

The release of 8-OH-dG in the supernatant from cell culture was measured by using the 8-OHdG (8-Hydroxydeoxyguanosine) ELISA Kit (Elabscience, E-EL-0028) according to the manufacturer’s protocol. Briefly, 50 µL cellular supernatant and standards were added to the microplate. Then immediately add 50 µL of Biotinylated Detection Ab working solution to each well and incubate for 45 min at 37°C. After three times of washing, add HRP Conjugate working solution and incubate for 30 min at 37°C. Wash five times and add 90 µL of substrate reagent to each well. After five times of washing, substrate reagent was added and incubated at 37°C for 15 min. Finally, the absorbance was measured at 450 nm after adding 50 µL of stop solution.

### Flow cytometric analysis

The purified CD19^+^ B cells were treated with 0.1 µg/mL mtDNA, 3 µg/mL CpG-2006 (Hycult Biotech, HC4039-200) or 0.5 µM iODN (InvivoGen, tlrl-4084) and mock-treated or infected with SFTSV (MOI 1–10) for 72 h. Subsequently, the cells were stained with the following fluorochrome-conjugated anti-human antibodies: PE-Cy7-conjugated anti-CD19, PerCP/Cy5.5 -conjugated anti-CD27, BV785-conjugated anti-CD38, PE-conjugated anti-CD62L, APC-conjugated anti-BAFFR, or PE-conjugated anti-human TLR9 for a duration of 30 min at 4°C. The antibodies are detailed in Table 2.

**TABLE 2:** Regents and resources used in this study

Reagent or resource	Source	Identifier
Antibodies		
PE-Cy7-conjugated anti-CD19	BioLegend	Cat# 302215
PerCP/Cy5.5-conjugated anti-CD27	BioLegend	Cat# 356407
BV785-conjugated anti-CD38	BioLegend	Cat# 303529
PE-conjugated anti-CD62L	BioLegend	Cat# 304805
APC-conjugated anti-BAFFR	BioLegend	Cat# 304805
P- conjugated anti-human TLR9	BioLegend	Cat# 394804
APC anti-human CD39 Antibody (clone A1)	BioLegend	Cat# 328210
PE anti-human CD62E Antibody (clone HCD62E)	BioLegend	Cat# 322605
Goat Anti-Mouse IgG antibody (FITC)	BioLegend	Cat# 405305
Caspase-3 Antibody	CST	Cat# 9662
Cleaved Caspase-3 (Asp175) Antibody	CST	Cat# 9661
Anti-DFNA5/GSDME Antibody	Abcam	Cat# ab215191
Mouse polyclonal anti-NP	This paper	
Virus strains		
SFTSV strain HBMC16_human_2015 (GenBank: KY440775.1, KY440776.1 and KY440777.1)	Wuhan Institute of Virology	PMID: 37531422
Biological samples		
Peripheral blood from healthy and SFTSV-infected adults	This paper	
Chemicals and peptides		
DMSO	Innochem	Cat# D3855
Ficoll-Paque plus	GE	Cat# 17144002
RBC Lysis Buffer	BioLegend	Cat# 420301
Mito-Tracker Red CMXRos	Beyotime	Cat# C1049B
MitoTracker Green FM	Invitrogen	Cat# M7514
MPTP Assay Kit	Beyotime	Cat# C2009S
MitoSOX Red	Invitrogen	Cat# M36008
CpG-2006	Hycult Biotech	Cat# HC4039-200
iODN	InvivoGen	Cat# tlrl-4084
Critical commercial assays		
Cytofix/Cytoperm Fixation/Permeabilization Solution Kit	BD Biosciences	Cat# 554714
CytoTox 96Non-Radioactive Cytotoxicity Assay	Promega	Cat# G1780
8-OHdG(8-Hydroxydeoxyguanosine) ELISA Kit	Elabscience	Cat# E-EL-0028
EasySep Direct Human Total Lymphocyte Isolation Kit	Stemcell Technologies	Cat# 17954
Mitochondria DNA Isolation kit	Abcam	Cat# ab65321
QIAmp DNA Blood Mini Kit	Qiagen	Cat# 51104
QIAamp MinElute Virus Spin Kit	Qiagen	Cat# 57704
TIANamp virus DNA/RNA kit	TIANGEN	Cat# DP315
One-step RT-PCR Kit	Takara	Cat# RR064A
Experimental models: cell lines		
Vero cells	ATCC	Cat# CCL81
Human umbilical vein endothelial cells	ATCC	Cat# PCS-100–010
Nalm-6	ATCC	Cat# CRL-3273
Raji	ATCC	Cat# CCL-86
H929	ATCC	Cat# CRL-3580
THP-1	ATCC	Cat# TIB-202
Oligonucleotides		
Primer: Mitochondrial NADH dehydrogenase 1 Forward: AGGACAAGAGAAATAAGGCC	Sangon Biotech	PMID: 30431116
Primer: Mitochondrial NADH dehydrogenase 1 Reverse: TAAGAAGAGGAATTGAACCTCTGACTGTAA	Sangon Biotech	PMID: 30431116
Probe: Mitochondrial NADH dehydrogenase 1 TTCACAAAGCGCCTTCCCCCGTAAATGA	Sangon Biotech	PMID: 30431116
Primer: Pax5 Forward: CTTGCTCATCAAGGTGTCAGGC	Sangon Biotech	PMID: 37751586
Primer: Pax5 Reverse: TGGCGACCTTTGGTTTGGATCC	Sangon Biotech	PMID: 37751586
Primer: IRF4 Forward: GAACGAGGAGAAGAGCATCTTCC	Sangon Biotech	PMID: 29367853
Primer: IRF4 Reverse: CGATGCCTTCTCGGAACTTTC	Sangon Biotech	PMID: 29367853
Primer: XBP1 Forward: CTGCCAGAGATCGAAAGAAGGC	Sangon Biotech	PMID: 32142632
Primer: XBP1 Reverse: CTCCTGGTTCTCAACTACAAGGC	Sangon Biotech	PMID: 32142632
TLR9-human siRNA: 5′- GGUGACCGUGCAGCCGGAGAUGUUUTT-3′	Sangon Biotech	PMID: 29880709
Negative control siRNA: 5′-UUCUCCGAACGUGUCACGUTT −3′	Sangon Biotech	PMID: 33795652
Primer: TLR9 Forward: ACTTCTTCCAAGGCCTGAGC	Sangon Biotech	PMID: 29880709
Primer: TLR9 Reverse: GGCCAGGTAATTGTCACGGA	Sangon Biotech	PMID: 29880709
Software and algorithms		
GraphPad Prism version 8	GraphPad Software	https://www.graphpad.com/
ImageJ	NIH, Univ. of Wisc. Madison	https://imagej.nih.gov/ij/
NovoExpress software	ACEC Biosciences	https://novoexpress.software.informer.com/

For the detection of intracellular SFTSV NP, the cells were initially fixed and permeabilized with a Fixation/Permeabilization Kit (BD, 554714), then incubated overnight with an SFTSV-NP antibody and subsequently stained with Goat Anti-Mouse IgG antibody (FITC) for 1 h at 4°C. The cells were washed twice using FACS solution and analyzed via flow cytometry.

HUVECs were seeded in 24-well plates to form a tight endothelial monolayer before being infected with SFTSV (MOI 1–10) for 72 h. Infected cells were collected and stained with APC anti-human CD39 Antibody (clone A1) as well as PE anti-human CD62E Antibody (clone HCD62E) for 1 h at 4°C. The resulting cell suspension was resuspended in PBS and analyzed through flow cytometry.

### Immunoblotting analysis

Cells were lysed using RIPA lysis buffer (Beyotime, P0013B) containing 1% PMSF (Thermofisher, 1861280) and phosphatase inhibitor mixture (Applygen, P1260) in ice. Protein concentration of the cell lysates was determined using a BCA Protein Quantification Kit (Vazyme, E112-01) according to the manufacturer’s instructions. Protein samples were heated at 100°C for 5 min in 5× SDS sample buffer. Protein lysates were separated by 10% or 12% SDS-polyacrylamide gel electrophoresis (PAGE) and then transferred to polyvinylidene difluoride (PVDF) membranes (Millipore, IPVH00010). To block non-specific binding sites, the membranes were incubated with Tris-buffered saline containing 0.05% Tween 20 (TBST) and 5% skim milk (BD Difco, 232100) for 1 h at room temperature. Following this step, the membranes were further incubated overnight at 4°C with the indicated primary antibodies, followed by horseradish peroxidase-conjugated secondary antibodies. Finally, protein bands were visualized using an enhanced chemiluminescence (ECL) kit (Vazyme, E412).

### RNA interference knockdown

The specific siRNAs were transfected into Nalm-6 cells with Lipofectamine RNAiMAX (Invitrogen, cat. 13778150). For 5  min, 25  pmol siRNA, and 2  µL RNAiMAX were incubated at room temperature, and the siRNA–lipid complex was subsequently added to the cells. In the parallel experiments, scrambled siRNA was included as a control. The efficiency of interference was determined by RT–qPCR. The specific siRNA for TLR9 was synthesized by Sangon Biotech, with sequences as listed in Table 2.

### Quantitative real-time PCR for B cell differentiation-related genes

Total RNA was extracted using the QIAamp MinElute Virus Spin Kit (QIAGEN, 57704). Assays-on-demand primers and probes and the TaqMan Universal Master Mix were used according to the manufacturer’s instructions. The quantitative real-time PCR (qRT-PCR) was performed with the One-step Primer Script RT-PCR kit (Takara, RR064A). The primers are detailed in Table 2.

### Human mtDNA sequencing

Mitochondrial DNA was isolated from cells using DNeasy Blood & Tissue Kits (Qiagen), quantified using the Qubit dsDNA HS Assay Kit (Sangon Biotech), and verified by 1% agarose gel electrophoresis. Full mitochondrial genomes were enriched via long-range PCR and processed into sequencing libraries using enzymatic fragmentation with Smearase, adapter ligation (Hieff NGS OnePot Pro DNA Library Prep Kit, Yeasen Biotechnology), and size selection (~400 bp, magnetic beads). Libraries were validated with Qubit 4.0 Fluorometer (Thermo Fisher Scientific) and 2% agarose gel before sequencing on Illumina NovaSeq XPlus (2 × 150 bp paired-end).

Raw data were processed with Fastp for adapter/quality trimming, aligned to rCRS using BWA-MEM, and deduplicated with SAMTools. Variants were called using GATK HaplotypeCaller, filtered with strict thresholds (QD <2, MQ <40, etc.), and annotated via ANNOVAR with mitochondrial databases (mtDB, MITOMAP).

### Quantification and statistical analysis

Continuous variables were described with means and standard deviations (SD) or medians and interquartile range (IQR). Categorical variables were described using frequencies and proportions. Student’s *t*-tests were employed to compare mean levels between two groups if the variable followed a normal distribution; otherwise, the Mann-Whitney U test was used. ANOVA or nonparametric tests were utilized for comparisons of continuous variables among multiple groups. χ^2^ tests or Fisher exact tests were employed to compare the percentages of categorical variables between groups. Log-binomial regression was applied to calculate the risk ratio (RR) and 95% confidence interval (CI) comparing the high cf-mtDNA copy group versus the low group. Kaplan-Meier analysis was conducted to determine survival associations. Scatter plots and correlation coefficients were used to assess the correlation between cf-mtDNA copy numbers and virus load. Additionally, GEEs were employed to compare dynamic changes in repeatedly measured laboratory indicators and viral loads between the two groups. Statistical analysis was performed using R version 3.6.3 (R Foundation for Statistical Computing, Vienna, Austria) as well as Stata 15.0 (Stata Corporation, College Station, TX, USA). A two-sided *P* < 0.05 was considered statistically significant.

## Data Availability

The data that support the findings of this study are available from the corresponding authors upon request.

## SUPPLEMENTAL MATERIAL

Fig. S1 jvi.01323-24-s0001.tif Flow diagram of patient selection.

Fig. S2 jvi.01323-24-s0002.tif Effect of mtDNA purified from non-infected HUVECs on B cells.

Fig. S3 jvi.01323-24-s0003.tif Effect of mtDNA purified from SFTSV-infected HUVECs on the proportion of SFTSV NP+ cells in THP-1 and 293T cells, and representative images of flow cytometry.

Fig. S4 jvi.01323-24-s0004.tif Mutation information of mtDNA of non-infected HUVECs and SFTSV-infected HUVECs.

ASM does not own the copyrights to Supplemental Material that may be linked to, or accessed through, an article. The authors have granted ASM a non-exclusive, world-wide license to publish the Supplemental Material files. Please contact the corresponding author directly for reuse.
