# Supplementary figures and images for: Endothelial cell-released mitochondrial DNA promotes B cell differentiation and virus replication during severe fever with thrombocytopenia syndrome virus infection
Source: J Virol. 2025 May 14;99(6):e01323-24. doi: 10.1128/jvi.01323-24 (PMC12172443; doi:10.1128/jvi.01323-24)

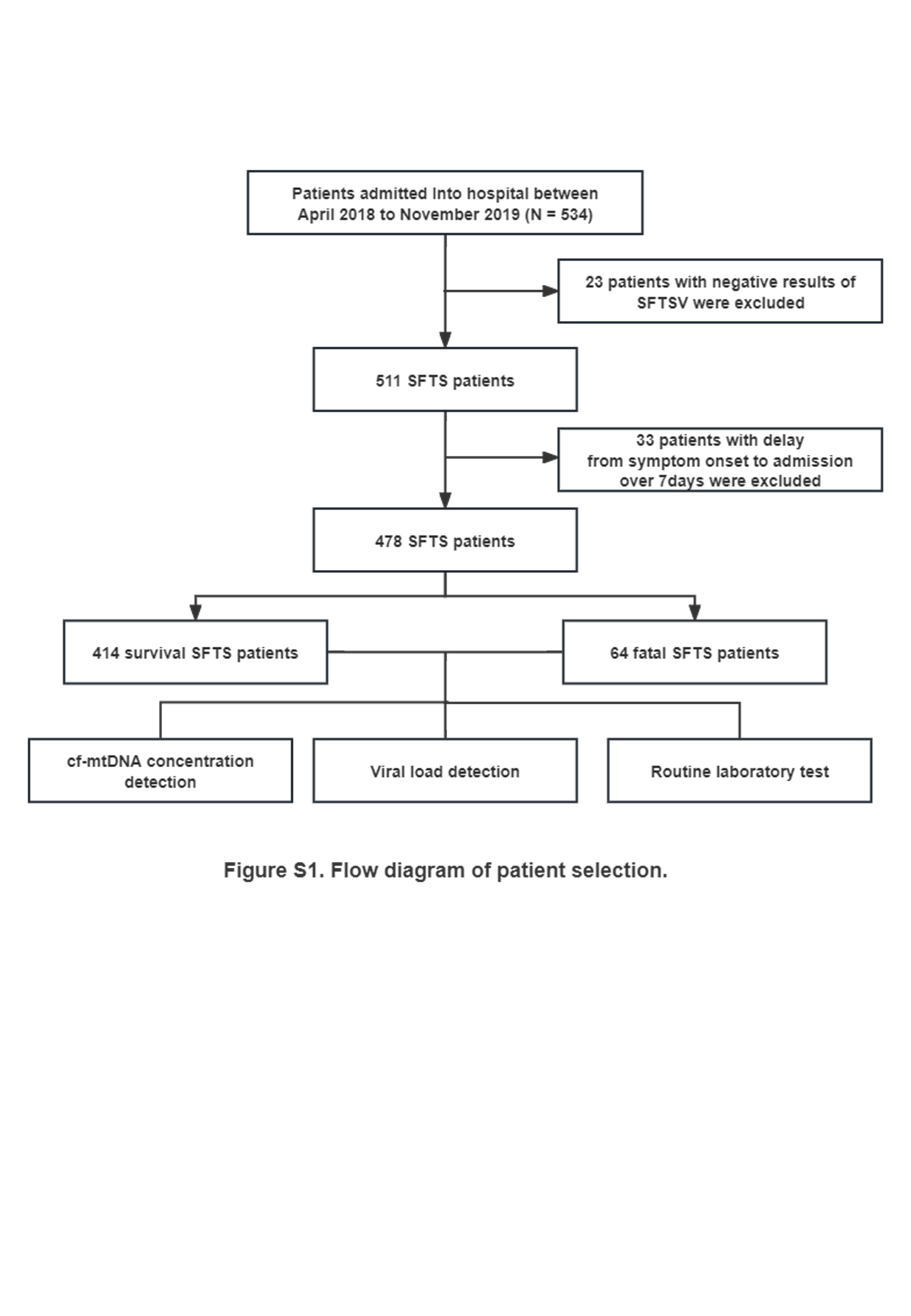

Supplement: Fig. S1 — Flow diagram of patient selection. [file jvi.01323-24-s0001.tif]

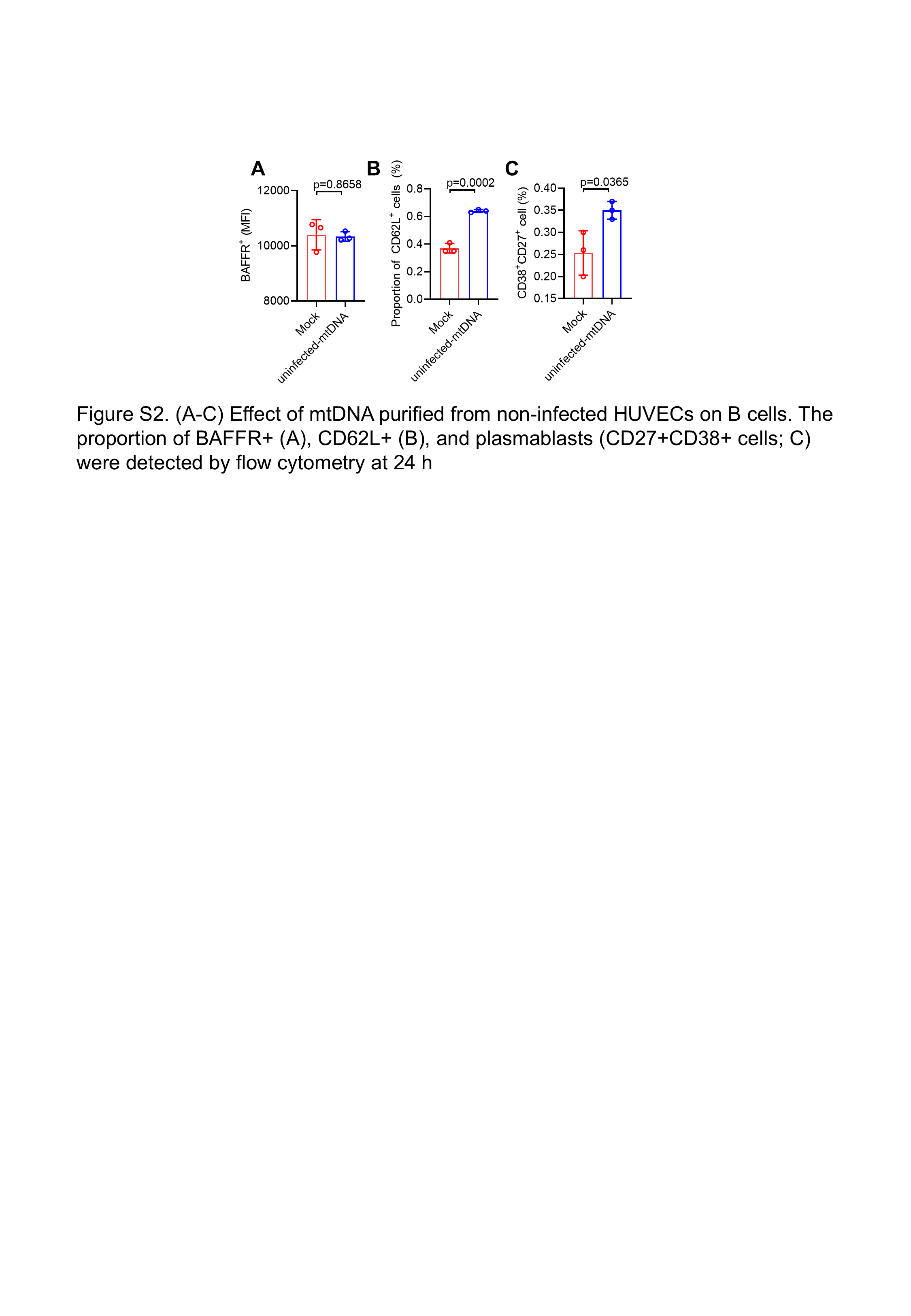

Supplement: Fig. S2 — Effect of mtDNA purified from non-infected HUVECs on B cells. [file jvi.01323-24-s0002.tif]

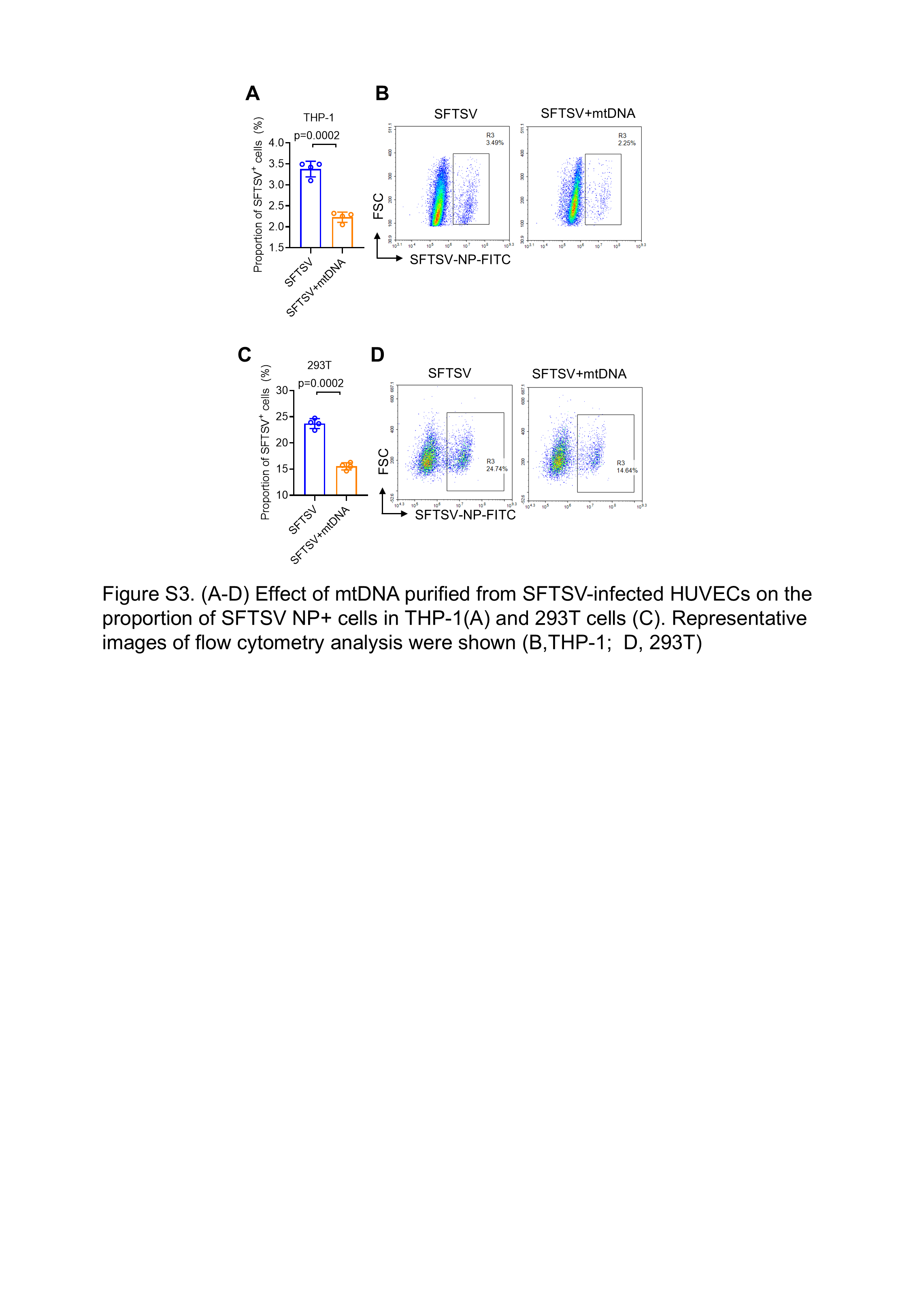

Supplement: Fig. S3 — Effect of mtDNA purified from SFTSV-infected HUVECs on the proportion of SFTSV NP+ cells in THP-1 and 293T cells, and representative images of flow cytometry. [file jvi.01323-24-s0003.tif]

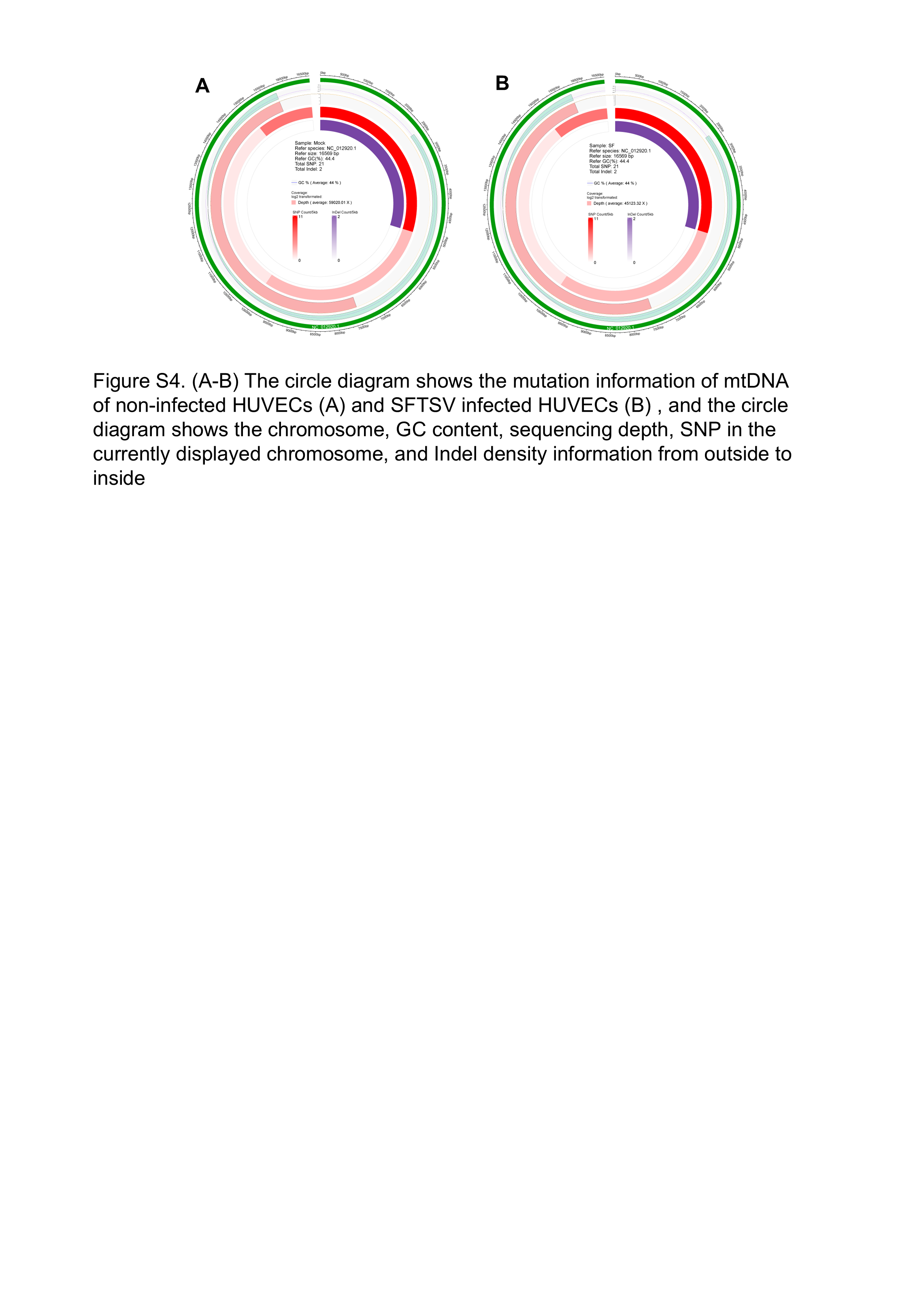

Supplement: Fig. S4 — Mutation information of mtDNA of non-infected HUVECs and SFTSV-infected HUVECs. [file jvi.01323-24-s0004.tif]
